# Neurodevelopment for syntactic processing distinguishes childhood stuttering recovery versus persistence

**DOI:** 10.1186/1866-1955-7-4

**Published:** 2015-01-20

**Authors:** Evan Usler, Christine Weber-Fox

**Affiliations:** Department of Speech, Language, and Hearing Sciences, Purdue University, Lyles-Porter Hall, 715 Clinic Drive, West Lafayette, IN 47907 USA

**Keywords:** Stuttering, Event-related potentials, Language processing, Language development, N400, P600, Children

## Abstract

**Background:**

Characterized by the presence of involuntary speech disfluencies, developmental stuttering is a neurodevelopmental disorder of atypical speech-motor coordination. Although the etiology of stuttering is multifactorial, language development during early childhood may influence both the onset of the disorder and the likelihood of recovery. The purpose of this study was to determine whether differences in neural indices mediating language processing are associated with persistence or recovery in school-age children who stutter.

**Methods:**

Event-related brain potentials (ERPs) were obtained from 31 6–7-year-olds, including nine children who do not stutter (CWNS), 11 children who had recovered from stuttering (CWS-Rec), and 11 children who persisted in stuttering (CWS-Per), matched for age, and all with similar socioeconomic status, nonverbal intelligence, and language ability. We examined ERPs elicited by semantic and syntactic (phrase structure) violations within an auditory narrative consisting of English and Jabberwocky sentences. In Jabberwocky sentences, content words were replaced with pseudowords to limit semantic context. A mixed effects repeated measures analysis of variance (ANOVA) was computed for ERP components with four within-subject factors, including condition, hemisphere, anterior/posterior distribution, and laterality.

**Results:**

During the comprehension of English sentences, ERP activity mediating semantic and syntactic (phrase structure) processing did not distinguish CWS-Per, CWS-Rec, and CWNS. Semantic violations elicited a qualitatively similar N400 component across groups. Phrase structure violations within English sentences also elicited a similar P600 component in all groups. However, identical phrase structure violations within Jabberwocky sentences elicited a P600 in CWNS and CWS-Rec, but an N400-like effect in CWS-Per.

**Conclusions:**

The distinguishing neural patterns mediating syntactic, but not semantic, processing provide evidence that specific brain functions for some aspects of language processing may be associated with stuttering persistence. Unlike CWS-Rec and CWNS, the lack of semantic context in Jabberwocky sentences seemed to affect the syntactic processing strategies of CWS-Per, resulting in the elicitation of semantically based N400-like activity during syntactic (phrase structure) violations. This vulnerability suggests neural mechanisms associated with the processing of syntactic structure may be less mature in 6–7-year-old children whose stuttering persisted compared to their fluent or recovered peers.

## Background

Developmental stuttering, often characterized by the presence of involuntary speech disfluencies such as speech sound repetitions, prolongations, and silent blocks, is a dynamic, multifactorial, neurodevelopmental disorder involving the coordination of speech motor processes [1–3]. This perspective of developmental stuttering assumes that stuttering onset is not linked to a single cause, but likely the outcome of interacting genetic, epigenetic, developmental, and environmental factors in early childhood. Although the etiological interaction between contributing factors is distinctive for every child who stutters, for a significant population, variables of language, such as semantic and syntactic processing, play a role in the breakdown of speech [4, 5].

The onset of developmental stuttering is most commonly reported around the late second and early third years of age [6, 7]. This suggests that stuttering may develop concurrently with critical stages of language acquisition [8], including an increase in cumulative vocabulary and lexical retrieval ability [9, 10], along with increasing use of multiple morpheme utterances [11]. The co-occurrence of stuttering onset and this rapid growth in semantic and syntactic abilities suggest that neural substrates mediating semantic and syntactic processing may be a factor in the emergence of stuttering and perhaps its chronicity.

Recent neuroimaging studies provide evidence of neuroanatomical and neurophysiological differences between children who stutter (CWS) and typically fluent children (CWNS) [12–18]. Using event-related brain potentials (ERPs), Weber-Fox and colleagues [18] observed significant differences in neural patterns mediating semantic and syntactic processing in 4–5-year-old CWS relative to fluent peers. Their findings, discussed in Electrophysiological indices of semantic and syntactic processing in stuttering section below, revealed more marked group differences for syntactic processing compared to semantic processing. It remains unclear if differences in language processing distinguish recovery from or persistence in stuttering. In the current study, we used ERPs to examine whether neural patterns mediating semantic and syntactic (phrase structure) processing distinguish stuttering persistence versus recovery in 6–7-year-old children.

### Behavioral indices of semantic and syntactic processing in CWS

The association between language and developmental stuttering has been studied extensively. Behavioral research using language assessments, measures of spontaneous language, and experimental measures, such as priming and reaction time analyses, has yielded complex and sometimes contradictory conclusions [19–21]. Some behavioral studies have reported linguistic abilities of CWS to be near or above those of CWNS [22–25]. Other studies have observed that CWS perform weaker on language tasks compared to CWNS [26, 27]. Similarly, a meta-analysis by Ntourou and colleagues [21] concluded that CWS differed significantly from CWNS in receptive vocabulary, expressive vocabulary, and mean length of utterance, with CWS generally performing relatively weaker. Subtle differences in lexico-semantic abilities have been recorded between CWS and CWNS [28–34]. CWS may also have greater difficulty perceiving and producing syntactic structure compared to CWNS [35–44].

Despite these reported differences in semantic and syntactic abilities between CWS and CWNS, behavioral research has been inconsistent in revealing distinguishing characteristics of linguistic ability associated with developmental stuttering. This is not surprising because language differences between CWS and CWNS are likely too subtle and heterogeneous to be uniformly characterized with behavioral assessments or standardized testing [4, 6]. This notion underscores the need for a direct, temporal analysis of the neural mechanisms underlying semantic and syntactic processing.

### Electrophysiological indices of semantic and syntactic processing in stuttering

By fitting electrodes over the scalp, electrical activity from the synchronous firing of pyramidal neuronal networks can be recorded [45]. The amplitude, latency, and distribution of these electroencephalographic (EEG) waveforms, time-locked to an external stimulus, can be averaged to reveal ERP components associated with aspects of language processing [46]. One widely recognized ERP index of semantic processing is the N400, a central-parietal negativity usually elicited between 200 and 600 ms after the onset of a stimulus, such as a semantic anomaly [47]. Correlated with the identification, retrieval, and integration of semantic meaning in long-term memory, N400 mean amplitude and peak latency have been associated with the level of expectation or cloze probability of a word within a semantic context [46, 47].

The P600 is a positive ERP component related to phrase structure repair during syntactic processing and is usually elicited by syntactic abnormalities, such as ungrammaticality and garden path sentences [48]. In adults, phrase structure violations typically elicit a biphasic waveform consisting of an early negativity component (~100–300 ms after stimulus onset) before a P600. This early negativity is typically seen in the anterior left hemisphere region and likely plays a role in the automatic construction of syntactic structure before the P600, which serves to later reanalyze and repair these structures in light of syntactic errors [49, 50].

ERP studies of adults who stutter (AWS) revealed atypical neural activity during semantic and syntactic processing relative to adults who do not stutter (AWNS), despite their having normal linguistic abilities [51–53]. In a natural speech processing paradigm, Weber-Fox and Hampton [53] observed semantic (verb expectation) and syntactic (verb agreement) violations in simple sentences respectively elicit typical N400 and P600 effects in AWNS. However, both semantic and syntactic violations elicited a similar biphasic pattern in AWS, consisting of a combined N400-P600 effect. In the only ERP study of semantic and syntactic processing in CWS, Weber-Fox and colleagues [18] observed significant differences between 4–5-year-old CWS and CWNS in neural activity mediating semantic and syntactic processing, despite having comparable socioeconomic status (SES), nonverbal IQ, and language abilities. Using stimuli identical to the current study, semantic violations elicited comparable N400 mean amplitude effects for both CWS and CWNS; however, CWS displayed a slightly longer N400 peak latency for both violation and control stimuli. Syntactic (phrase structure) violations also elicited a larger early negativity in CWS. While both groups displayed a P600 for phrase structure violations relative to canonical sentences, this effect was significant over the left hemisphere for CWNS and over the right hemisphere for CWS. Their findings suggest the relative patterns of neural activity for processing syntax may be atypical in preschool-age CWS.

### Using Jabberwocky to investigate syntactic processing

To parse semantic and syntactic aspects of language processing, numerous studies have presented subjects with Jabberwocky sentence stimuli [54–59]. The use of Jabberwocky sentences allows one to observe neural activity associated with syntactic processing with reduced semantic context by replacing meaningful content words with phonologically valid pseudowords [60]. The appearance of Jabberwocky influences the characteristics of ERP components elicited during language processing in both adults and children. Yamada and Neville [54] found syntactic violations elicited a robust early negativity (~200 ms), but a weaker P600 effect in Jabberwocky compared to English sentences. A similar study also found a weaker P600 elicited in Jabberwocky sentences relative to English over the left hemisphere [55]. A study of 3-year-old typically developing children reported a late negativity (600–700 ms), instead of a P600, elicited by phrase structure violations within Jabberwocky sentences [56]. In a similar study with 3-year-olds, Silva-Pereyra and colleagues [57] observed a P600-like effect elicited by phrase structure violations within English sentences, but the elicitation of late negativities (750–1,150 ms) without a P600 for Jabberwocky. More recently, Yamada and colleagues observed a late negativity elicited by phrase structure violations within Jabberwocky in typically developing 3–5-year-olds [personal communication]. In these ERP studies of syntactic processing in young children, the observed negativities during Jabberwocky sentences are more N400-like, with a later and more posteriorly located elicitation than the early left anterior negativity seen in typical adults [49, 50]. The elicitation of a component similar to the N400, a neural activity associated with semantic processing, suggests that young children may use semantically based strategies for the comprehension of syntax when presented with Jabberwocky sentences. These ERP findings are consistent with behavioral evidence that suggest young children rely on semantic cues for sentence comprehension before the development of mature syntactic processing in later childhood [61–66].

### Distinguishing children who persist from those who recover from stuttering

Researchers have sought to identify linguistic variables associated with stuttering recovery versus persistence [14, 67–70]. Chang and colleagues [14] found that 9–12-year-old CWS with persistent stuttering had decreased fractional anisotropy in white matter underlying the left rolandic operculum compared to children who had previously recovered. The purpose of our study was to determine if differences in neural correlates mediating semantic and syntactic processing are associated with persistence or recovery in young school-age children who stutter. We hypothesized that ERP indices of syntactic processing, but not semantic processing, would likely distinguish stuttering persistence versus recovery. Our expectation was based on previous findings that ERP differences during semantic processing were less marked than those elicited during syntactic processing in younger 4–5-year-old CWS compared to fluent peers [18] and that N400 maturation is robust across populations varied in language competency [71]. Although atypical ERP activity has been observed in AWS by Weber-Fox and Hampton [53], their auditory semantic verb expectation task likely required more demanding linguistic processing compared to the processing of semantic noun violations in our current study. Thus, we did not expect ERP patterns for semantic processing to distinguish recovery from persistence of stuttering.

## Methods

### Participants

The 31 6–7-year-old children in this study were participants in the longitudinal Purdue Stuttering Project beginning when they were 4–5 years old, at which time participants from both the current CWS-Per and CWS-Rec groups exhibited developmental stuttering. A spontaneous speech-language sample, half of which was collected during parent-child interaction and half during examiner-child interaction, was recorded during the participant’s first year in the project to help determine CWS classification. Recruited from the local community, these children also participated in a study using a different but similar paradigm 2 years previously [18]. Included were nine children with normal fluency (CWNS; one female), 11 children who had recovered from stuttering at the time of testing (CWS-Rec; two females), and 11 children who persisted in stuttering at the time of testing (CWS-Per; three females).

Participants were evaluated based on the criteria established by Yairi and Ambrose to determine whether or not they demonstrated developmental stuttering [72, 73]. A child was regarded as exhibiting developmental stuttering if he/she was identified as such by the speech-language pathologist who worked on the project and a parent, if stuttering severity was rated as two or higher on an eight-point severity scale (0–7) by the speech-language pathologist or a parent, and if the child exhibited at least three stuttering-like disfluencies per hundred syllables of spontaneous speech according to Yairi and Ambrose’s weighted index [73]. Part-word repetitions, monosyllabic repetitions, and dysrhythmic phonations such as silent blocks and sound prolongations were categorized as stuttering-like disfluencies. After this initial classification at 4–5 years of age, subsequent speech-language samples were collected annually to help determine if the participants met the criteria for persistence or recovery.

Previous research suggested that SES may be a significant influence on cognitive and language abilities [74, 75]. Hollingshead’s education scale [76] was used to evaluate the level of maternal education as a measure of participant SES. Mean (standard error) SES scores for the CWNS, 6.56 (.18); CWS-Rec, 6.09 (.28); and CWS-Per, 5.45 (.31) differed significantly [*F*(2,28) = 3.93, *p* = .03]. Although there was a difference in SES between CWNS and CWS-Per [*F*(1,18) = 8.34, *p* = .01], there was no difference between CWS-Per and CWS-Rec [*F*(1,20) = 2.27, *p* = .15] nor between CWNS and CWS-Rec [*F*(1,18) = 1.73, *p* = .21]. Further discussion concerning group differences in SES is provided in the Study limitations section (see Tables 1 and 2 for participant characteristics, including age, SES, family history, stuttering characteristics, and history of therapy).

**Table 1:** **Participant characteristics and test scores**

Participant	Age	Sex	Handedness	Age of onset	Family history of stuttering	Stuttering characteristics	Stuttering index at 4–5	Stuttering index at 6–7	Severity at 4–5	Severity at 6–7	Stuttering Tx	Other Tx	SES	CMMS	TACL-3	SPELT-3	BBTOP-Cl
CWNS-1	6;1	M	R	NA	No	NA	NA	NA	NA	NA	No	No	7	119	139	119	103
CWNS-2	7;10	M	R	NA	No	NA	NA	NA	NA	NA	No	No	7	116	126	114	89
CWNS-3	6;8	F	R	NA	No	NA	NA	NA	NA	NA	No	No	6	119	121	117	117
CWNS-4	7;1	M	R	NA	No	NA	NA	NA	NA	NA	No	No	6	122	121	106	100
CWNS-5	6;4	M	R	NA	No	NA	NA	NA	NA	NA	No	No	6	119	109	102	111
CWNS-6	7;7	M	R	NA	No	NA	NA	NA	NA	NA	No	No	6	105	113	109	101
CWNS-7	7;9	M	R	NA	No	NA	NA	NA	NA	NA	No	No	7	120	106	120	101
CWNS-8	6;11	M	L	NA	No	NA	NA	NA	NA	NA	No	No	7	104	124	100	118
CWNS-9	7;3	M	R	NA	No	NA	NA	NA	NA	NA	No	No	7	121	141	127	113
Mean													6.556	116.111	122.2	112.667	105.889
SD													0.527	6.791	12.13	9.028	9.506
SE													0.176	2.264	4.044	3.009	3.169
CWS Rec-1	6;9	M	R	3;6	No	SS, PW	7.87	0.63	2.5	1	No	No	6	111	139	100	104
CWS Rec-2	6;2	M	R	2;6	No	SS, PW	2.99	0.1	3	0	Yes	No	6	103	104	104	104
CWS Rec-3	7;9	M	R	2;0	No	SS, PW	6	1.89	3	1.5	No	No	6	100	117	100	105
CWS Rec-4	7;7	F	R	3;0	Yes	SS, PW, DP	5.36	1.38	4.25	1.5	Yes	No	5	104	100	94	86
CWS Rec-5	7;8	M	R	2;6	No	SS, DP	4.04	1.03	2.5	1	No	No	7	118	121	110	89
CWS Rec-6	7;10	M	L	2;0	Yes	SS, PW	16.89	1.78	5	1.5	Yes	No	7	100	117	102	107
CWS Rec-7	7;3	M	R	3;0	Yes	SS, PW, DP tension	3.18	0.8	3	1	No	No	4	107	109	102	110
CWS Rec-8	6;2	M	R	2;6	Unknown	SS, PW, DP	3.14	2.29	3	1.5	No	No	6	105	117	117	92
CWS Rec-9	6;7	M	R	3;0	Yes	SS, PW	3.38	2.41	3.5	0	Yes	A	7	121	111	102	80
CWS Rec-10	7;9	M	R	3;0	No	SS, PW	2.21	1.05	2	0	No	No	6	117	111	102	99
CWS Rec-11	6;3	F	R	2;0	No	SS, PW, DP	7.83	1.49	3.5	1.5	No	No	7	103	111	92	111
Mean							5.99	1.35	3.227	0.955			6.091	108.091	114.3	102.273	98.818
SD							4.087	0.713	0.876	0.65			0.944	7.503	10.24	6.813	10.458
SE							1.232	0.215	0.264	0.196			0.285	2.262	3.087	2.054	3.153
CWS Per-1	6;10	M	R	3;0	No	SS, PW, DP	18.93	6.56	5.5	2.5	Yes	No	4	110	113	103	104
CWS Per-2	6;1	M	R	3;0	No	SS, PW, DP tension	7.73	8	4	4.5	No	No	6	112	117	100	87
CWS Per-3	7;5	M	R	4;0	No	SS, PW, DP	10.27	8.29	4.5	4	Yes	No	6	118	113	106	99
CWS Per-4	6;11	M	R	3;8	Yes	SS, PW, DP tension	18.93	28.95	5.5	5.5	Yes	No	5	122	119	103	100
CWS Per-5	6;11	F	R	3;0	Yes	SS, PW, DP tension	4.46	4.46	3.5	3.5	Yes	No	6	137	104	100	100
CWS Per-6	7;6	M	R	4;0	No	SS, PW, DP	10.19	18.41	5	5.5	Yes	A	4	122	111	95	95
CWS Per-7	6;0	M	R	3;0	Yes	SS, PW, DP tension	26.52	9.19	6	5	Yes	A	7	112	121	90	89
CWS Per-8	7;1	M	R	4;0	No	SS, PW, DP	16.25	1.65	4.5	3	No	No	6	132	132	121	103
CWS Per-9	6;5	F	R	2;0	Yes	SS, PW, DP tension	6.58	9.55	4.5	3	Yes	No	6	108	111	100	93
CWS Per-10	7;2	F	R	2;6	Unknown	SS, PW, DP	39.82	2.4	6	2	Yes	A	4	116	126	98	85
CWS Per-11	6;2	M	R	2;0	No	SS, PW	9.39	3.13	4	2	No	No	6	115	109	105	113
Mean							15.37	9.145	4.818	3.682			5.455	118.545	116	101.909	97.091
SD							10.448	8.042	0.845	1.309			1.036	9.136	8.075	7.803	8.312
SE							3.15	2.425	0.255	0.395			0.312	2.755	2.435	2.353	2.506

**Table 2:** **Group mean and standard errors for age, socioeconomic status, nonverbal IQ, and language assessment scores**

	CWNS	CWS-Rec	CWS-Per	Group statistics
Age	7.05 (.21)	7.07 (.13)	6.77 (.16)	*F*(2,28) = .78, *p* = .47
SES	6.56 (.18)	6.09 (.29)	5.45 (.31)	*F*(2,28) = 3.93, *p* = .03*
CMMS	116.11 (2.26)	108.09 (2.26)	118.55 (2.75)	*F*(2,28) = 5.16, *p* = .01*
TACL-3	122.22 (4.04)	114.27 (3.09)	116 (2.43)	*F*(2,28) = 1.64, *p* = .21
SPELT-3	112.67 (3.01)	102.27 (2.05)	101.91 (2.35)	*F*(2,28) = 5.80, *p* = .01*
BBTOP-CI	105.89 (3.17)	98.82 (3.15)	97.09 (2.51)	*F*(2,28) = 2.34, *p* = .12

Note: *SES* socioeconomic status, *CMMS* Columbia Mental Maturity Scale, *TALC-3* Test for Auditory Comprehension of Language-Third Edition, *SPELT-3* Structured Photographic Expressive Language Test-Third Edition, *BBTOP-CI* Bankson-Bernthal Test of Phonology-Consonant Inventory. *p < .05.

Note: Age of onset provided by parent; stuttering characteristics = single syllable whole word (SS), part-word repetition (PW), dysrhythmic phonation (DP); stuttering index = # weighted of SLDs/100 syllables [see 73], evaluated at initial visit (4–5 years of age) and 6–7 years of age; severity = stuttering severity rating (0–7) provided by clinician, evaluated at initial visit (4–5 years of age) and 6–7 years of age; stuttering Tx = exposure to therapy of stuttering before or during time of testing at 6–7 years of age; other Tx = exposure to other therapies, such as for articulation (A), before or during time of testing at 6–7 years of age; SES = socioeconomic status [see 76 for scale]; CMMS = Columbia Mental Maturity Scale; TACL-3 = Test for Auditory Comprehension of Language-Third Edition; SPELT-3 = Structured Photographic Expressive Language Test-Third Edition; BBTOP-CI = Bankson-Bernthal Test of Phonology-Consonant Inventory.

The CWNS and CWS-Rec groups each contained one left-handed participant, while the CWS-Per group did not contain any left-handed participants, confirmed by an abbreviated handedness inventory [77]. According to parental report, all of the participants were native-English speaking, had normal or corrected-to-normal vision, and no history of neurological disorders. No participants had a history of taking medications, with the exception of one child in the CWS-Rec group with a history of medication for attention-deficit hyperactivity disorder. All participants demonstrated normal hearing, as confirmed by a hearing screening at 20 dB HL for 500, 1,000, 2,000, 4,000, and 6,000 Hz. No participants demonstrated symptoms of impaired reciprocal social interaction and restriction of activities [78] as assessed by the Childhood Autism Rating Scale [79]. Normal nonverbal intelligence was demonstrated by all participants as assessed by the Columbia Mental Maturity Scale (CMMS) [80]. As displayed in Table 2, there was a significant difference between groups for the CMMS [*F*(2,28) = 5.16, *p* = .01]. CWS-Rec performed slightly lower compared to CWNS [*F*(1,18) = 6.15, *p* = .02] and CWS-Per [*F*(1,20) = 8.60, *p* = .01], but there was no difference in CMMS between CWNS and CWS-Per [*F*(1,18) = .44, *p* = .52]. Discussion of group differences in CMMS is provided in the Study limitations section.

A battery of tests was administered to all of the participants to assess phonological abilities, expressive language, and receptive language proficiencies. The Test for Auditory Comprehension of Language-Third Edition (TACL-3) [81] measured language comprehension, the Structured Photographic Expressive Language Test-Third Edition (SPELT-3) [82] assessed spoken language, and the Consonant Inventory subtest of the Bankson-Bernthal Test of Phonology (BBTOP-CI) [83] measured phonological abilities. On each of these assessments, all participants scored within or above normal limits, with the exception of one CWS-Rec participant with a slightly below-average BBTOP-CI score. Participant scores on the TACL-3 [*F*(2,28) = 1.64, *p* = .21] and BBTOP-CI [*F*(2,28) = 2.34, *p* = .12] were comparable across groups. However, between-group performance on the SPELT-3 was significantly different [*F*(2,28) = 5.80, *p* = .01]. There were differences between CWNS and CWS-Per [*F*(1,18) = 8.18, *p* = .01] and between CWNS and CWS-Rec [*F*(1,18) = 8.62, *p* = .01]. Performance between CWS-Per and CWS-Rec was not different [*F*(1,20) = .01, *p* = .91]. Again, a discussion concerning group differences in SPELT-3 is provided in the Study limitations section. Group mean scores for the TACL-3, SPELT-3, and BBTOP-CI are presented in Table 2.

### Sentence stimuli

In collaboration with the Brain Development Lab in Eugene, Oregon, directed by Dr. Helen Neville, sentence stimuli were developed to accompany visual displays of cartoon videos of “Pingu” the penguin. The monitor displayed the ongoing cartoon with a visual angle of 5° horizontally and 4° vertically. Using Presentation software (9.70), the visual display was accompanied with naturally spoken sentences presented via a speaker placed directly above the monitor at an average intensity of 70–75 dB SPL. Different versions of these Pingu stimuli were previously used in a study of language processing in younger children [18], many of whom participated in the current study.

During the ERP recording session, participants watched five cartoon videos, each consisting of 100 naturally spoken sentences and approximately 7–8 min in duration. All of the words used to form the sentence stimuli were taken from the MacArthur Communicative Developmental Inventories: Words and Sentences [84] to ensure that the sentences were comprised of vocabulary familiar to the participants. The sentences were spoken by male and female speakers at a natural rate. Three trained researchers at the Brain Development Laboratory in Eugene, Oregon, made independent judgments to determine the onsets of the canonical and violation words.

A total of five linguistic constraints were quasi-randomly included in the stimuli, and ERPs elicited by three of those constraints were analyzed for the current study. Semantic violations were included in 50 sentences. An example of a sentence with a semantic violation includes “Mommy waves her *snow* goodbye.” Syntactic (phrase structure) violations were embedded in 50 English sentences and 50 Jabberwocky sentences. An example of an English sentence with a phrase structure violation is “He wants to play with those *his* toys.” Jabberwocky sentences were created by replacing the content words within the corresponding English sentence with phonologically valid pseudowords and included syntactic phrase structure violations identical to those in the English sentences. For example, a Jabberwocky sentence with a phrase structure violation is “Ho digbay to tangwon with those *his* bowz”. Having identical syntactic violations within both English and Jabberwocky sentences allowed us to observe the influence of the presence and absence of semantic context on participants’ syntactic processing. Each of these 150 violation sentences had a corresponding control sentence. Two scenarios were developed for each cartoon video so that a sentence that served as a violation condition in one scenario (e.g. story #2A, “The music box is on the *name* so Pingu can push it.”) would serve as the control sentence in another scenario (e.g. story #2B “The music box is on the *sled* so Pingu can push it.”). Examples of sentences for each condition are shown in Table 3. The remaining 200 sentences, whose data is not included, consisted of irregular-verb agreement and regular-verb agreement conditions.

**Table 3:** **Examples of the sentence stimuli for each condition**

Sentence type	Example sentences
Semantic canonical	She closes her *door* in pingu.
	Daddy is holding another *present* for pingu.
	The play a *game* in the snow.
	Pingu is building a *castle* on the floor.
	Pingu wants to play *music*, too.
Semantic violation	She closes her *head* on pingu.
	Daddy is holding another *backyard* to pingu.
	They play a *hand* in the snow.
	Pingu is building a *music* on the floor
	Pingu wants to play *hat,* too.
Phrase structure canonical (English)	Mommy and Daddy look at *their* son.
	He makes lots of noise with *that* accordion.
	Pingu sits on top of *this* igloo.
	Pinga walks back to *her* sled withher head down.
	Pingu chews with *his* mouth open.
Phrase structure violation (English)	Mommy and Daddy look at that *their* son.
	He makes lots of noise with this *that* accordion.
	Pingu sits on top of their *this* igloo.
	Pinga walks back to their *her* sled with her head down.
	Pingu chews with that *his* mouth open.
Phrase structure canonical (Jabberwocky)	Moonoo and dobah hokee at *their* sim.
	Zhay pangdoom trayglee of toopem with *that* apelgoeem.
	Kamgi trahbahn on top of *this* ubre.
	Pantue boshveen back to *her* aheep with her burmar peem.
	Fue reeab with *his* sheepum okim.
Phrase structure violation (Jabberwocky)	Moonoo and dobah hokee at that *their* sim.
	Zhay pangdoom trayglee of toopem with this *that* apelgoeem.
	Kamgi trahbahn on top of their *this* ubre.
	Pantue boshveen back to their *her* aheep with her burmar peem.
	Fue reeab with that *his* sheepum okim.

### Procedures

Before the presentation of the stimuli, children were seated 60 in. in front of an 18.5-in. monitor within a sound-attenuating booth. An experimenter sat with the child and gave the following instructions: “While you sit in this chair, you will watch and listen to five stories about Pingu the penguin and his family and friends. It is important to keep your arms, legs, and head as still as you can while you are watching the stories. At the end of each story, you will have a break where you can move and stretch if you need to. You will also get to pick out a sticker and place it on your activity sheet at the end of each story. When you have five stickers on your sheet, you will be finished and will get to pick out a toy!” The accompanying experimenter helped the child with picking out a sticker after each story and helped the child remain still during the video presentations.

### Electroencephalographic recording

Electroencephalogram (EEG) signals were recorded from the scalp with an elastic electrode cap (Quick-cap, Compumedics-Neuroscan). Consistent with the international 10-10 system [85], the cap contained 32 Ag-Cl electrodes positioned in homologous locations, including the lateral sites F7/F8, FT7/FT8, T7/T8, TP7/TP8, and P7/P8; the medial sites FP1/FP2, F3/F4, FC3/FC4, C3/C4, CP3/CP4, P3/P4, and O1/O2; and the midline sites FZ, FCZ, CZ, CPZ, PZ, and OZ. To monitor horizontal eye movements, bipolar recordings obtained from electrodes were placed on the left and right outer canthi. Eye blinks were monitored with bipolar recordings from electrodes placed on the left superior and inferior orbital ridges. Electrodes were placed on the left and right mastoids to serve as an online reference. Recordings were re-referenced offline to an average of the electrode recordings from the left and right mastoid placements [45]. Electrode impedances were adjusted to ≤10 kΩ for VEOG and HEOG channels and ≤5 kΩ for all other electrode sites. The EEG was digitized online at a rate of 500 Hz and band-pass filtered between 0.1 and 100 Hz.

### ERP analyses

Movement artifacts, including eye blinks, were removed from EEG activity using independent component analysis (ICA) and automatic artifact rejection algorithms using EEGLAB [86]. The use of ICA allowed us to isolate components reflecting eye movement and other artifacts unrelated to language processing and remove these components from our analysis. EEG waveforms were then low-pass filtered at 30 Hz, and time-locked epochs between -200 and 2,000 ms were created. Trials were averaged by condition for each participant using ERPLAB [87]. There were no significant group differences in the number of trials accepted across conditions [*F*(10,140) = 1.64, *p* = .14]. Group means of accepted trials for each condition are summarized in Table 4.

**Table 4:** **Means and standard errors for trials accepted for semantic and syntactic conditions**

Group	Semantic condition		Syntactic condition (English)		Syntactic condition (Jabberwocky)	
	Canonical	Violation	Canonical	Violation	Canonical	Violation
CWNS	37.33 (1.29)	37.22 (1.53)	32.67 (1.99)	34.44 (1.32)	30.78 (1.43)	34.78 (1.55)
CWS-Rec	37.18 (1.17)	38 (1.41)	37.64 (1.09)	34 (1.58)	30.91 (1.44)	33.09 (1.15)
CWS-Per	38.27 (1.14)	38.09 (1.54)	36.27 (1.22)	35.09 (1.18)	33.45 (.85)	34.64 (1.49)

**Figure 1:**
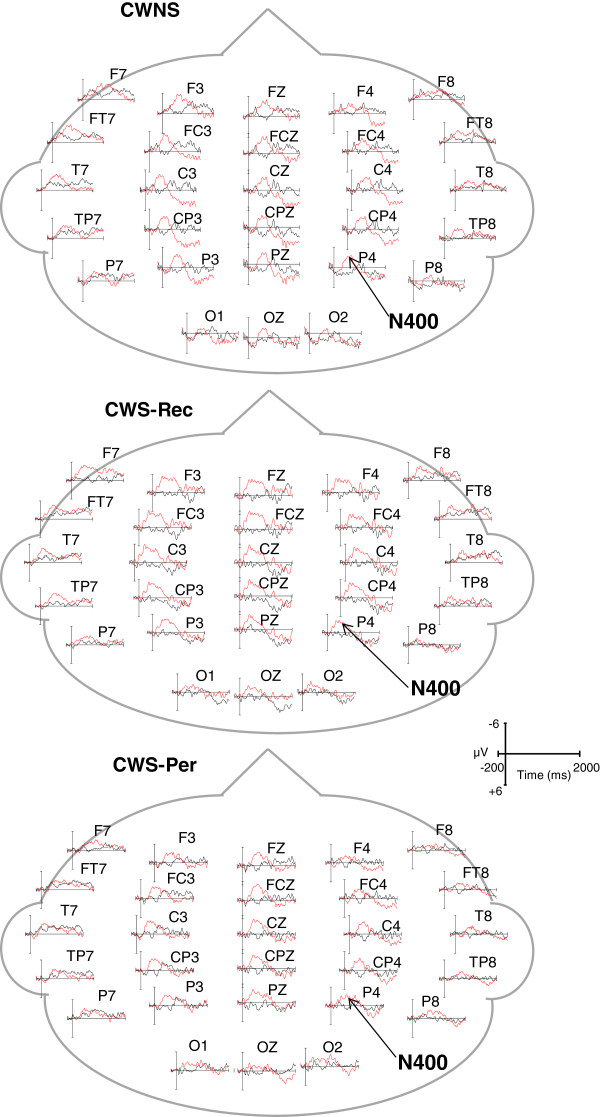
**Grand average ERPs elicited by the semantic condition.** ERPs of all participants in the CWNS, CWS-Rec, and CWS-Per groups reveal similar N400 waveforms elicited in the semantic canonical (black) and violation (red) conditions.

**Figure 2:**
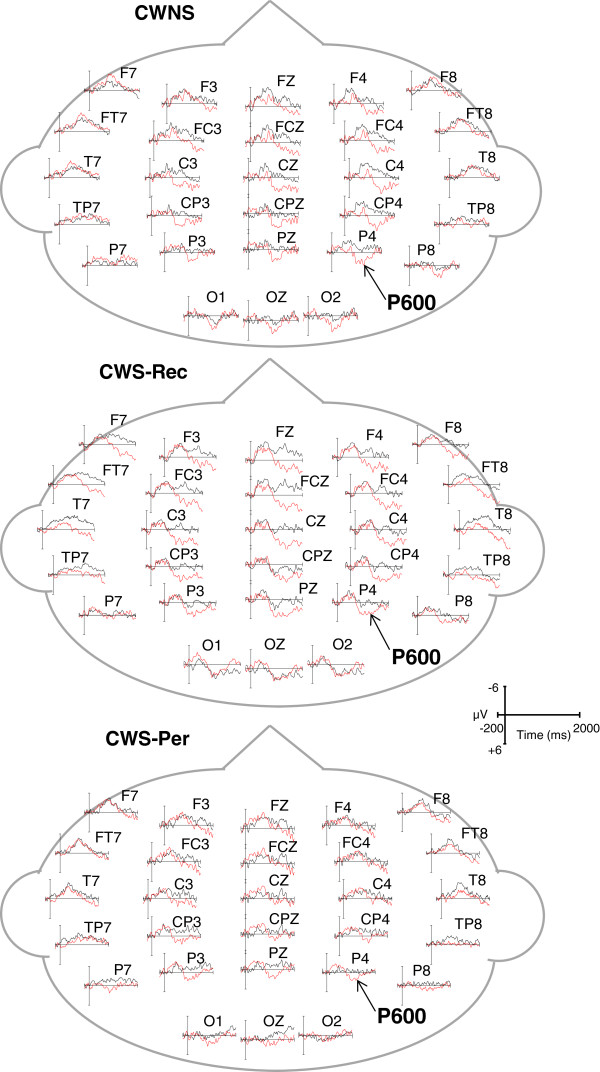
**Grand average ERPs elicited by the syntactic condition within English sentences.** ERPs of all participants in the CWNS, CWS-Rec, and CWS-Per groups reveal similar P600 waveforms elicited in the English syntactic canonical (black) and violation (red) conditions.

**Figure 3:**
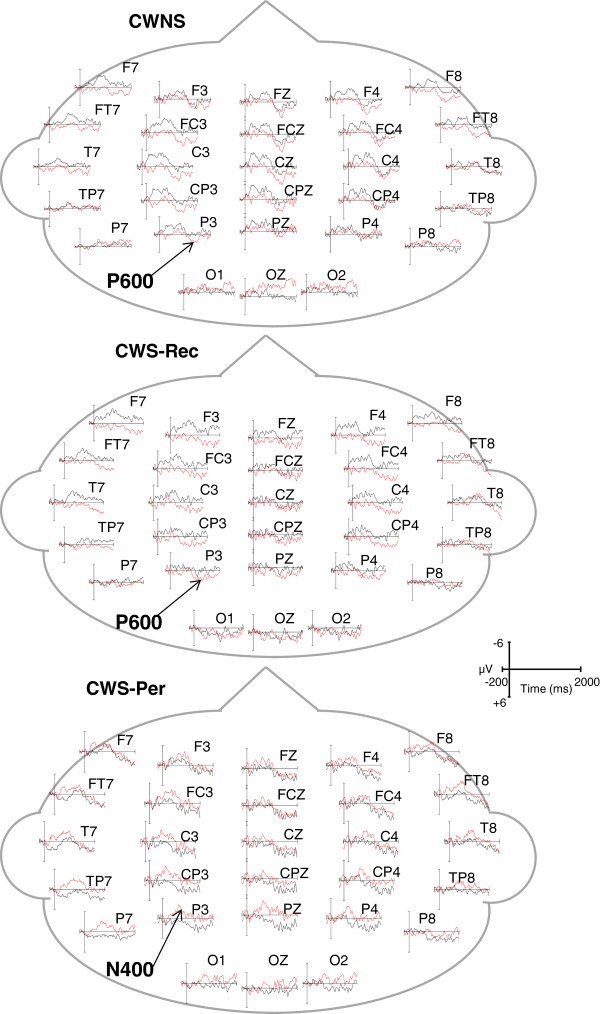
**Grand average ERPs elicited by the syntactic condition within Jabberwocky sentences.** ERPs of all participants in the CWNS, CWS-Rec, and CWS-Per groups, showing waveforms elicited in the Jabberwocky syntactic canonical (black) and violation (red) conditions.

In order to measure individual ERP components, temporal windows were selected by centering the temporal windows on the peaks of each component in the grand averages and consistent with an earlier ERP study of semantic and syntactic processing in CWS by Weber-Fox et al. [18]. In the semantic condition, mean amplitude and peak latency of N400 were measured using a 450–750-ms temporal window. For the phrase structure condition, windows of 150–300 and 600–900 ms were used to measure syntactic negativities in English and Jabberwocky. P600 mean amplitudes were measured using a temporal window of 1,000–1,300 ms for English sentences and a 1,200–1,500-ms window for Jabberwocky sentences. The later temporal window for Jabberwocky compared to that for English was based on the previous finding that the elicitation of the P600 for processing syntactic violations within Jabberwocky is attenuated compared to the P600 elicited by identical violations within English [54]. For display purposes, the ERP waveforms shown in Figures 1, 2, and 3 were low-pass filtered at 20 Hz.

**Figure 4:**
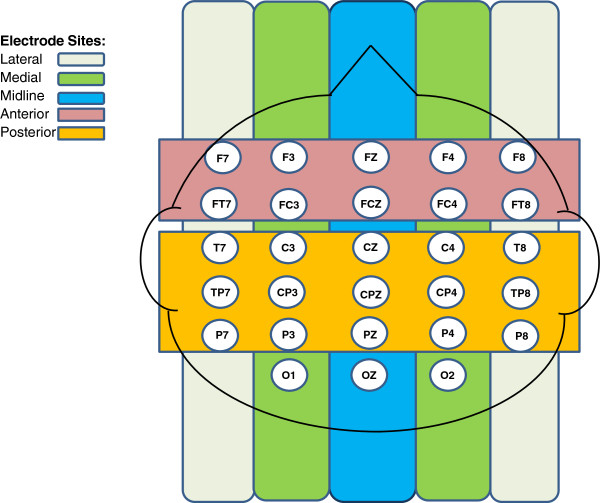
**Scalp map consisting of approximate locations for all electrodes included in analyses.**

A mixed effects repeated measures analysis of variance (ANOVA) was computed for each of the ERP components with four within-subject factors including condition (canonical, violation), hemisphere (left, right), anterior/posterior distribution (anterior: frontal, fronto-central; posterior: central, central-parietal, parietal), and laterality (lateral, medial). Group (CWNS, CWS-Rec, CWS-Per) served as a between-subject factor. Condition (canonical, violation) and anterior/posterior distribution (anterior: FZ, FCZ; posterior: CZ, CPZ, PZ) factors were also computed for the midline electrodes. Figure 4 displays all electrodes used in our analyses. To provide more direct analyses between CWS-Rec and CWS-Per, repeated measures ANOVA with just these two groups were also performed. Significant differences in these component measures were considered using an alpha level of *p* < .05. Huynh-Feldt adjusted *p* value was used if the degree of freedom of the numerator was greater than 1 [88].

## Results

### ERP indices of semantic processing

#### N400 (450–750 ms)

Grand average ERPs elicited by canonical (black) and semantic violation (red) conditions are displayed for CWNS, CWS-Rec, and CWS-Per (Figure 1). A condition effect was observed at lateral/medial electrodes, with each group displaying an N400 with a significantly greater mean amplitude elicited by semantic violations compared to canonical conditions [*F*(1,28) = 35.53, *p* < .001]. A similar condition effect was seen for midline electrodes [*F*(1,28) = 35.36, *p* < .001]. A group × condition effect for N400 mean amplitude was not found at lateral/medial electrodes [*F*(2,28) = .19, *p* = .83] nor midline electrodes [*F*(2,28) = .02, *p* = .98]. In regard to N400 peak latency, no condition effect was observed at lateral/medial electrodes [*F*(1,28) = .30, *p* = .59] nor at midline electrodes [*F*(1,28) = .01, *p* = .94]. Furthermore, no group × condition effect for peak latency was found for lateral/medial electrodes [*F*(2,28) = .42, *p* = .66] nor for midline electrodes [*F*(2,28) = .78, *p* = .47]. When comparing just CWS-Rec and CWS-Per, no group × condition effect at lateral/medial electrodes was observed for N400 mean amplitude [*F*(1,20) = .39, *p* = .54]. There were also no group × condition effect for midline electrodes [*F*(1,20) = .009, *p* = .926].

### ERP indices of syntactic processing

#### Phrase structure violations within English sentences

##### Syntactic negativities (150–300/600–900 ms)

Grand average ERPs elicited by canonical (black) and syntactic violation (red) conditions are displayed for CWNS, CWS-Rec, and CWS-Per (Figure 2). Across groups, no condition effects were observed for either the early or later negativities. Syntactic (phrase structure) violations within English sentences did not elicit a significantly different early negativity (150–300 ms) component mean amplitude relative to the canonical condition at lateral/medial electrodes [*F*(1,28) = .02, *p* = .90]. A condition effect for the early negativity mean amplitude did not reach significance for midline electrodes [*F*(1,28) = 3.82, *p* = .06]. There were no group × condition effects for the early negativity mean amplitude at lateral/medial electrodes [*F*(2,28) = .20, *p* = .82] and at midline electrodes [*F*(2,28) = .25, *p* = .78]. Additionally, a comparison of CWS-Rec and CWS-Per revealed no group × condition effects for mean amplitude of the early negativity for lateral/medial electrodes [*F*(1,20) = .43, *p* = .52] and for midline electrodes [*F*(1,20) = .70, *p* = .41].

A later syntactic negativity (600–900 ms) was also not different for the violation relative to the canonical condition at lateral/medial electrodes [*F*(1,28) = 3.92, *p* = .58] nor at midline electrodes [*F*(1,28) = 1.55, *p* = .22]. There was no group × condition effect for the later negativity mean amplitude for lateral/medial electrodes [*F*(2,28) = .23, *p* = .80] nor for midline electrodes [*F*(2,28) = 1.24, *p* = .31]. Again, comparing CWS-Rec and CWS-Per revealed no group × condition effects for mean amplitude of the later negativity at lateral/medial electrodes [*F*(1,20) = .38, *p* = .55] and midline electrodes [*F*(1,20) = .85, *p* = .37].

#### P600 (1,000–1,300 ms)

A condition effect characterized by a bilateral robust P600 was elicited in all three groups relative to the canonical condition for lateral/medial electrodes [*F*(1,28) = 31.84, *p* < .001] and midline electrodes [*F*(1,28) = 28.78, *p* < .001] (Figure 2). There were no group × condition effects in P600 mean amplitude at lateral/medial electrodes [*F*(2,28) = 2.64, *p* = .09] nor at midline electrodes [*F*(2,28) = 1.65, *p* = .21]. A group × condition effect including just the CWS-Rec and CWS-Per groups did not reach significance for lateral/medial electrodes [*F*(1,20) = 4.11, *p* = .06]. For midline electrodes, a group × condition × AP effect was observed [*F*(5,100) = 2.63, *p* = .048]. However, further step-down ANOVAs failed to reach significant group × condition effects at anterior midline electrodes [*F*(1,20) = 2.48, *p* = .13] and at posterior midline electrodes [*F*(1,20) = 4.33, *p* = .051].

#### Phrase structure violations within Jabberwocky sentences

##### Syntactic negativities (150–300/600–900 ms)

Grand average ERPs elicited by canonical (black) and syntactic violation (red) conditions are displayed for CWNS, CWS-Rec, and CWS-Per (Figure 3). Similar to the English sentences, no condition effects were observed for either the early or later negativities. Within Jabberwocky sentences, syntactic (phrase structure) violations did not elicit a significant early negativity (150–300 ms) relative to the canonical condition for lateral/medial electrodes [*F*(1,28) = 1.03, *p* = .32] nor for midline electrodes [*F*(1,28) = 1.79, *p* = .19]. There were also no group × condition effects for the early negativity mean amplitude for lateral/medial electrodes [*F*(2,28) = 2.87., *p* = .07] nor for midline electrodes [*F*(2,28) = 1.91, *p* = .17]. A comparison of CWS-Rec and CWS-Per revealed no group × condition effects for mean amplitude of the early negativity for lateral/medial electrodes [*F*(1,20) = 3.06, *p* = .10] and for midline electrodes [*F*(1,20) = 1.03, *p* = .32].

**Figure 5:**
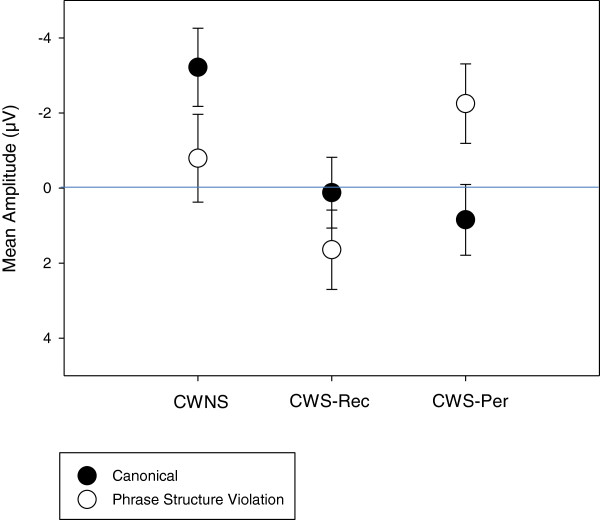
**N400-like mean amplitude for the Jabberwocky syntactic condition.** Group mean amplitude (μV) (600–900 ms) elicited by syntactic (phrase structure) violations within Jabberwocky sentences. Averaged across central-parietal electrodes (C3, CP3, P3, CZ, CPZ, PZ, C4, CP4, and P4), only CWS-Per show a larger negativity (N400-like effect) elicited by phrase structure violations relative to the canonical (negative plotted up).

**Figure 6:**
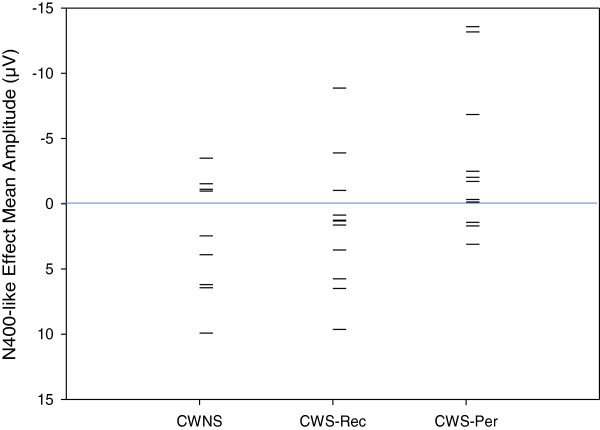
**N400-like effect distribution for the Jabberwocky syntactic condition.** Scatterplot showing the distribution of mean amplitude (600–900 ms) differences (phrase structure violations minus canonical phrase structures) within Jabberwocky sentences, averaged across central-parietal electrodes (C3, CP3, P3, CZ, CPZ, PZ, C4, CP4, and P4). This distribution reveals the individual differences within the CWNS, CWS-Rec, and CWS-Per groups. Each datum represents a single child (negative plotted up).

There was no condition effect for the later (600–900 ms) negativity at medial/lateral electrodes [*F*(1,28) = .93, *p* = .34] nor at midline electrodes [*F*(1,28) = .00, *p* = .99]. However, a group × condition effect, as graphed in Figure 3, revealed differences between groups in the elicitation of a later negativity (600–900 ms) at lateral/medial electrodes [*F*(2,28) = 6.45, *p* = .01], but not midline electrodes [*F*(2,28) = 1.88, *p* = .17]. Step-down ANOVAs revealed differences between CWNS and CWS-Per [*F*(1,18) = 11.16, *p* = .004] and between CWS-Rec and CWS-Per [*F*(1,20) = 9.19, *p* = .01], but not between CWNS and CWS-Rec [*F*(1,18) = .01, *p* = .94]. Figure 5 displays a group N400-like mean amplitude averaged across central-parietal electrodes. Relative to the canonical condition, phrase structure violations elicited a negativity only for CWS-Per. However, as graphed on Figure 6, significant overlap exists in ERP elicitation across participants. This reveals considerable individual differences in neural patterns, including N400-like activity, elicited over central-parietal sites. Still, no positive activity over 5 microvolts (μV) was elicited by phrase structure violations for any of the CWS-Per participants, unlike numerous CWNS and CWS-Rec participants.

#### P600 (1,200–1,500 ms)

**Figure 7:**
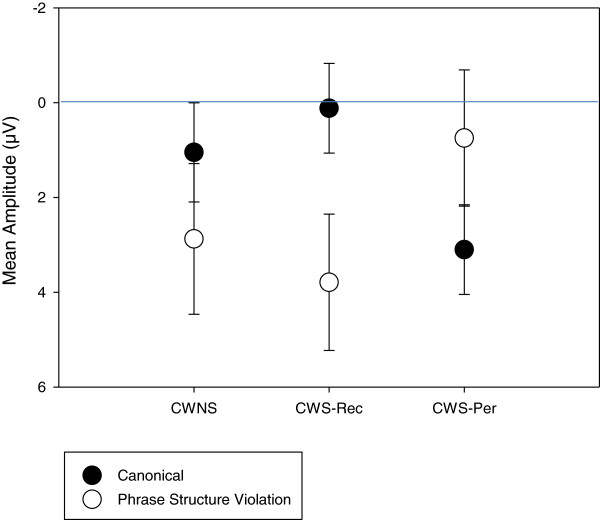
**P600 mean amplitude for the Jabberwocky syntactic condition.** Group mean amplitude (μV) (1,200–1,500 ms) elicited by syntactic (phrase structure) violations within Jabberwocky sentences. Averaged across central-parietal electrodes (C3, CP3, P3, CZ, CPZ, PZ, C4, CP4, and P4), only CWNS and CWS-Rec show a larger positivity (P600) elicited by phrase structure violations relative to the canonical condition (negative plotted up).

**Figure 8:**
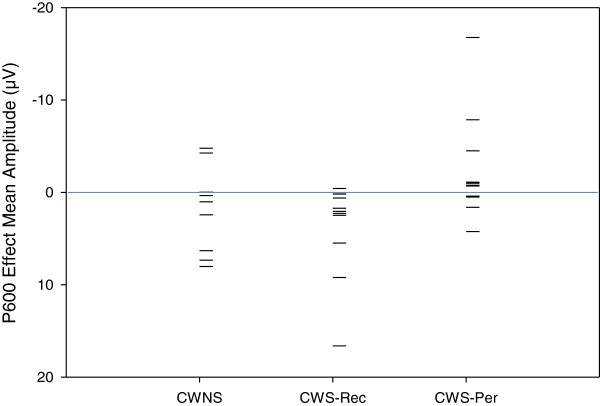
**P600 effect distribution for the Jabberwocky syntactic condition.** Scatterplot showing the distribution of mean amplitude (1,200–1,500 ms) differences (phrase structure violations minus canonical phrase structures) within Jabberwocky sentences, averaged across central-parietal electrodes (C3, CP3, P3, CZ, CPZ, PZ, C4, CP4, and P4). This distribution reveals the individual differences within the CWNS, CWS-Rec, and CWS-Per groups. Each datum represents a single child (negative plotted up).

In a later interval, a condition effect was not observed for all three groups relative to the canonical condition for lateral/medial electrodes [*F*(1,28) = 1.87, *p* = .18] and midline electrodes [*F*(1,28) = .91, *p* = .35]. However, a P600 was observed at lateral/medial electrodes for CWNS and CWS-Rec, resulting in a group × condition effect [*F*(2,28) = 4.36, *p* = .02]. The group × condition effect was not significant for midline electrodes [*F*(2,28) = 2.13, *p* = .14]. P600 mean amplitude was similar between CWNS and CWS-Rec [*F*(1,18) = .65, *p* = .43]. However, a group × condition effect was observed between CWNS and CWS-Per [*F*(1,18) = 4.69, *p* = .04] and between CWS-Rec and CWS-Per [*F*(1,20) = 8.19, *p* = .01]. Unlike the other two groups, the phrase structure violation stimuli in Jabberwocky did not elicit a P600 relative to the canonical in CWS-Per (Figure 7). However, individual differences were also observed within groups regarding ERP elicitation during this interval. As graphed in Figure 8, P600 mean amplitudes at central-parietal electrodes reveal considerable individual differences among the participants.

## Discussion

We examined ERPs elicited by semantic and syntactic (phrase structure) violations within an auditory narrative of English and Jabberwocky sentences to determine whether neural activity associated with semantic and syntactic processing distinguishes 6–7-year-old CWNS, CWS-Rec, and CWS-Per. Although all of these children previously participated in an earlier study regarding stuttering and language processing during the early preschool years [18], variables of persistence and recovery in later years were not addressed in this earlier study. In our current study, semantic violations elicited a qualitatively similar N400 in all three groups. Phrase structure violations within English sentences elicited a qualitatively similar P600 in all groups. Within Jabberwocky sentences, however, these same violations elicited an N400-like activity in CWS-Per, distinguishing this group from CWNS and CWS-Rec, who exhibited similar P600s.

### ERP activity mediating semantic processing did not distinguish school-age CWS-Per, CWS-Rec, and CWNS

Our findings indicate that neural activity mediating semantic processing does not distinguish CWS-Per from CWS-Rec and CWNS. Semantic violations elicited a qualitatively similar N400 component across groups. These findings are consistent with a previous study using identical stimuli that found no differences in N400 mean amplitude in 4–5-year-old CWS and CWNS [18]. In this previous study, however, a later N400 peak latency was observed in CWS compared to fluent controls, suggesting that preschool-age CWS had relatively slower, slightly less efficient semantic processing. This less mature pattern was not seen in the current study, which is not surprising given the N400 decreases in latency in later childhood [46]. At 6–7 years of age, CWS-Per and CWS-Rec participants may have overcome this semantic immaturity.

The view that semantic processing is not substantially deficient in CWS [6, 19, 20] is confirmed by these findings. Furthermore, they support the arguments by Bernstein Ratner [4] and Bloodstein [89] that it is unlikely lexico-semantic factors play a significant role in the disorder because developmental stuttering typically emerges after the development of early vocabulary and the use of simple word combinations. The qualitative similarities in the N400 between groups may be associated with the early development of semantic processing [90, 91]. Studies have observed children as young as 14 months exhibiting N400-like activity when presented with audio-visual semantic incongruities [92] and distinguishing phonologically similar versus dissimilar nonsense words [93]. This development occurs before the typical period of stuttering onset around 30–36 months [6, 7]. Furthermore, the N400 is less sensitive to maturational constraints during childhood, allowing a typical N400 component to develop even as late as early adolescence [71]. Given these characteristics, it is not surprising that the N400 does not distinguish our groups.

Still, our findings run counter to evidence of atypical semantic processing in AWS [53] and behavioral evidence of differences in the lexico-semantic abilities of CWS compared to CWNS [21, 29–34]. These differences are likely due to differences in the semantic stimuli used in these other tasks. Regarding ERP evidence of atypical semantic processing in AWS, the semantic verb expectation task given by Weber-Fox and Hampton [53] was likely more linguistically demanding compared to the semantic stimuli we presented. In addition, participants were asked to make overt grammaticality/semantic judgments in the Weber-Fox and Hampton study which may have increased the processing strategy and load. It should also be noted that the majority of studies regarding the semantic abilities of CWS, including the studies mentioned above, consisted of preschool-age participants. It is possible that by 6–7 years of age, CWS-Rec and CWS-Per in the current study may have caught up to their fluent peers in regard to semantic processing of relatively simple semantic noun violations.

### ERP activity mediating syntactic (phrase structure) processing of English sentences did not distinguish school-age CWS-Per, CWS-Rec, and CWNS

The processing of phrase structure is typically characterized by an early negativity and P600 component in adults [49, 50]. In our study, none of the groups exhibited a significant early negativity. This observation differed from a previous finding that 4–5-year-old CWS produced a significantly larger early negativity compared to CWNS [18]. Although this discrepancy may be explained by Weber-Fox and colleagues’ use of a different baseline (-1,000 to 2,500 ms), it is not surprising given conflicting evidence regarding the presence of an early negativity component in children [94, 95].

Within English sentences, phrase structure violations elicited a broadly distributed and bilateral P600 in all groups, suggesting similar syntactic repair processes during comprehension. This finding was unexpected given Weber-Fox et al.’s [18] observation that preschool-age CWS exhibited a P600 reduced over the left hemisphere and increased over the right hemisphere relative to fluent peers. A reduced and narrowly distributed P600 was also reported in AWS compared to fluent controls [51]. Although the language skills of the AWS group were within normal limits, this group performed worse than controls on linguistic assessments. Hampton Wray and Weber-Fox [96] highlighted that varieties in language proficiencies, even within the normal range, have an effect on the neural mechanisms associated with syntactic processing. Similarly, it was found that typical adults with relatively lower English proficiency exhibited a reduced P600 compared to their more highly proficient peers [97]. Our current findings differed from these previous studies, as we observed that all three groups exhibited qualitatively similar neural activity facilitating syntactic processing of simple English sentences. Furthermore, the morphology of the P600s elicited in CWS-Rec and CWNS appears consistent with P600 waveforms of typically developing 6- and 7-year-old children elicited by syntactic phrase structure violations [58].

### ERP activity mediating syntactic (phrase structure) processing of Jabberwocky sentences distinguishes school-age CWS-Per from CWS-Rec and CWNS

In addition to English sentences, we examined ERPs elicited by phrase structure violations within Jabberwocky sentences in the three groups. A similar P600 effect was elicited relative to the canonical condition in CWS-Rec and CWNS. This neural activity was comparable to the P600 elicited during the English sentences and to elicited waveforms of typically developing 6- and 7-year-olds in a study by Hahne and colleagues [58]. The lack of a traditional biphasic early negativity-P600 pattern in these children during the processing of English and Jabberwocky sentences was expected because the neural mechanisms underlying syntactic processing are still maturing [98, 99]. It is evident that for these two groups, the presentation of pseudowords in place of content words did not appear to significantly disturb phrase structure processing. However, the same cannot be said for CWS-Per, who did not exhibit a P600, but an N400-like effect. Because CWS-Per exhibited a P600 during English sentences, the striking N400-like effect observed during Jabberwocky is probably due to the lack of semantic cues that this group requires for sentence comprehension. To the contrary, CWS-Rec and CWNS were able to process syntactic structure without the aid of semantic cues.

Our findings are congruent with theories of language acquisition that describe the use different cues, including lexico-semantic cues, event probability, and word order heuristics, for sentence comprehension before the maturation of robust syntactic processing [60–65]. Hirsh-Pasek and Golinkoff [62] have shown that young children (~24–36 months) in early stages of grammatical development require a coalition of correlated semantic, syntactic, prosodic, and environmental cues to successfully comprehend simple sentences. Although syntactic skills develop throughout childhood and into adulthood [98, 99], the ability to rely on syntactic cues without the aid of semantic context for simple sentence comprehension typically emerges around 3 years of age [63, 66]. CWS-Per lacked this robust syntactic ability despite being 6–7 years of age. Instead, the elicitation of an N400-like effect by syntactic violations implies that CWS-Per rely on semantic cues to comprehend syntax, similar to younger, typically developing children before mature grammatical development.

The use of immature or compensatory sentence comprehension strategies has been seen in elderly native-speaking adults [100, 101], patients with Parkinson’s disease [102], and children with specific language impairment [103]. Kotz and colleagues [104] observed that patients with left temporal-parietal lesions exhibited a P600 while patients with basal ganglia lesions exhibited N400-like activity while listening to syntactic verb argument structure violations in German sentences. Interestingly, developmental stuttering has been associated with reduced structural and functional connectivity within the basal ganglia-thalamocortical network [15]. Novice, low-proficiency adult second language (L2) learners also exhibited an N400 effect in response to syntactic violations, while a P600 was elicited in native speakers and advanced L2 learners [105–107]. Tanner and colleagues [107] suggested the development of L2 proficiency may include a transition from a novice reliance on semantic processing (indexed by an N400) to more mature, rule-based syntactic processing (indexed by a P600) which is typically seen in native and advanced L2 speakers.

The syntactic immaturity of CWS-Per may be explained by recent neuroanatomical and neurophysiological findings regarding the development of syntactic processing in children. Nuñez and colleagues [99] have shown that increased syntactic ability is correlated with increased specialization of the inferior frontal gyrus in the left hemisphere. This region is one of a number of cortical areas critical for the processing of syntax, connected by white matter pathways in the form of dorsal and ventral processing streams [108–112]. Numerous studies have reported that the at least one dorsal stream, involving the arcuate fasciculus, facilitates complex syntactic processing in adults [112, 113]. In contrast, less mature syntactic processing by typically developing preschool-age children has been associated with increased activation in a ventral stream consisting of the extreme capsule fiber system [114, 115].

In both children and adults who stutter, neuroanatomical and neurophysiological anomalies have been observed along ventral and dorsal streams [12, 14, 15]. Chang and colleagues [14] reported 9–12-year-old children with persistent stuttering had attenuated functional left dorsal stream connectivity in the arcuate fasciculus compared to children who had recovered. More recently, Chang and Zhu [15] reported reduced functional connectivity in the left extreme capsule fiber system, which facilitates the ventral stream, in 3–9-year-old CWS compared to fluent controls. We speculate that a reduction in functional connectivity due to immaturity or inefficiency of dorsal and ventral streams critical for syntactic processing may be associated with the group differences in ERP activity observed in our current study.

### Implications and future directions

Given the distinguishing N400-like elicitation in CWS-Per to phrase structure violations within Jabberwocky, we assume 1) without adequate semantic cues, some children with persistent stuttering may be less efficient in processing the phrase structure of simple sentences compared to their recovered or fluent peers and 2) because of this syntactic inefficiency, these children may process sentence components not according to syntactic rules but as distinct lexical items. On the other hand, CWS-Rec were distinguished from their persisting peers by their more mature, robust use of syntactic cues.

The maturational difference between semantic and syntactic processing observed in CWS-Per gives evidence that a dissociation or imbalance between linguistic processing domains may characterize the neurophysiology of stuttering [26, 27]. A developmental asynchrony between more advanced semantic and less advanced syntactic abilities was previously correlated with increased speech disfluency in preschool-age children with developmental language disorders [116, 117].

For speech language pathologists and other professionals who provide therapeutic treatment, it is extremely important to identify distinguishing characteristics associated with future persistence or recovery to efficiently allocate limited resources to those most likely to persist. Although developmental stuttering has a naturally high recovery rate, a specific population of these children are resistant to natural recovery and may later suffer from severe physical and psychosocial consequences if stuttering develops into adulthood, including avoidance behaviors, anxiety disorders, societal and employment disadvantages, and decreased overall quality of life [118–120].

Future research with children closer to typical age of onset (~ages 2–5) using a similar experimental paradigm would be beneficial to determine if these same differences in syntactic processing exist. Future findings could possibly elucidate a neural signature for stuttering recovery and the value of language processing for predicting future stuttering persistence versus recovery. Although the findings from this study were not predictive, the older age of the participants in this study is significant, as the 6–7-year-old age range is associated not only with decreasing ability to acquire native-level syntactic competence [121, 122] but also with the end of a critical period for likely recovery from stuttering [6].

### Study limitations

Some variables that were not controlled for this study include participants’ exposure to speech therapy, the duration since stuttering onset, and the duration since recovery for CWS-Rec. Although unlikely, participants in the CWS-Per group may recover in later years [6]. The likelihood of recovery declines with increasing years post-stuttering onset [73]. Six out of the 11 CWS-Per participants were diagnosed with stuttering at least 3 years before testing. Four others were diagnosed 2 years before testing, while one participant was diagnosed 1 year prior. Due to these durations since diagnosis, the likelihood of recovery among our 6–7-year-old participants is minimal.

Group differences in SES, CMMS, and SPELT-3, reported in the Methods section, were further explored by including these variables as covariates in our ANOVA. Regarding our finding of group differences in P600 mean amplitude for Jabberwocky sentences, no condition effects were observed for SES [*F*(1,23) = .30, *p* = .59], CMMS [*F*(1,23) = .52, *p* = .48], and SPELT-3 [*F*(1,23) = 1.17, *p* = .29]. Similarly, for the group differences in N400-like mean amplitude for Jabberwocky sentences, no condition effects were seen for SES [*F*(1,23) = .12, *p* = .74], CMMS [*F*(1,23) = .22, *p* = .64], and SPELT-3 [*F*(1,23) = 1.53, *p* = .23]. The lack of condition effects, the observation of similar ERP activity for semantic or syntactic violation conditions within English sentences across groups, and participant scores within or above normal limits all suggest that the group differences reported in the Methods section regarding SES, CMMS, and SPELT-3 likely did not play a role in our main ERP findings.

## Conclusions

The purpose of this study was to determine whether neural patterns mediating semantic and syntactic processing during comprehension of an auditory narrative distinguished stuttering persistence versus recovery in young school-age children. ERPs obtained from 6–7-year-olds, including those with persistent stuttering, those who recovered from stuttering, and fluent controls, revealed differences in the neural correlates of syntactic processing despite all participants having normal language abilities. During comprehension of English sentences, neural indices mediating semantic and syntactic processing did not distinguish the three groups. Semantic violations elicited a qualitatively similar N400 across groups, while phrase structure violations in English and Jabberwocky elicited a similar P600 in CWNS and CWS-Rec. Although the neural indices of CWS-Per were comparable to the other two groups during English comprehension, removing semantic context affected the syntactic processing of CWS-Per. The resulting N400-like activity elicited by phrase structure violations, which typically meditates semantic recall and integration, is evidence that children with persistent stuttering employed an atypical, immature semantic strategy for processing phrase structure instead of using syntactic rules like their naturally fluent and recovered peers.

### Consent

Written informed consent was obtained from the participant’s legal guardian(s) following guidelines established by the institutional review boards of the participating institution and for publication of this research. A copy of the written consent is available for review by the Editor-in-Chief of this journal.
